# P-1610. Low-Dose Aripiprazole is a Promising Drug for Long COVID Treatment: A Retrospective Observational Study

**DOI:** 10.1093/ofid/ofaf695.1788

**Published:** 2026-01-11

**Authors:** Jason Cui, Muge Kalaycioglu, Giorgio Camillo Ricciardiello Mejia, Mehr Grewal, Tom Quach, Vincent Xin, Amogha Paleru, Anita Chopra, Linda Geng, Hector F Bonilla

**Affiliations:** Stanford University School of Medicine, Stanford, California; Stanford University, Palo Alto, California; Stanford University, Palo Alto, California; University of Washington, Bellevue, Washington; Stanford University, Palo Alto, California; Stanford University, Palo Alto, California; Stanford University, Palo Alto, California; University of Washington, Bellevue, Washington; Stanford Medicine, Stanford, California; Stanford University School of Medicine, Stanford, California

## Abstract

**Background:**

Long COVID (LC) is defined as persistence of new symptoms (Sx) for > 12 weeks following SARS-CoV-2, affecting multiple organ systems. There is no approved drug for LC treatment. We report an observational retrospective study on off-label use of low-dose Aripiprazole (LDA) for managing LC symptoms.

**Figure 1.**
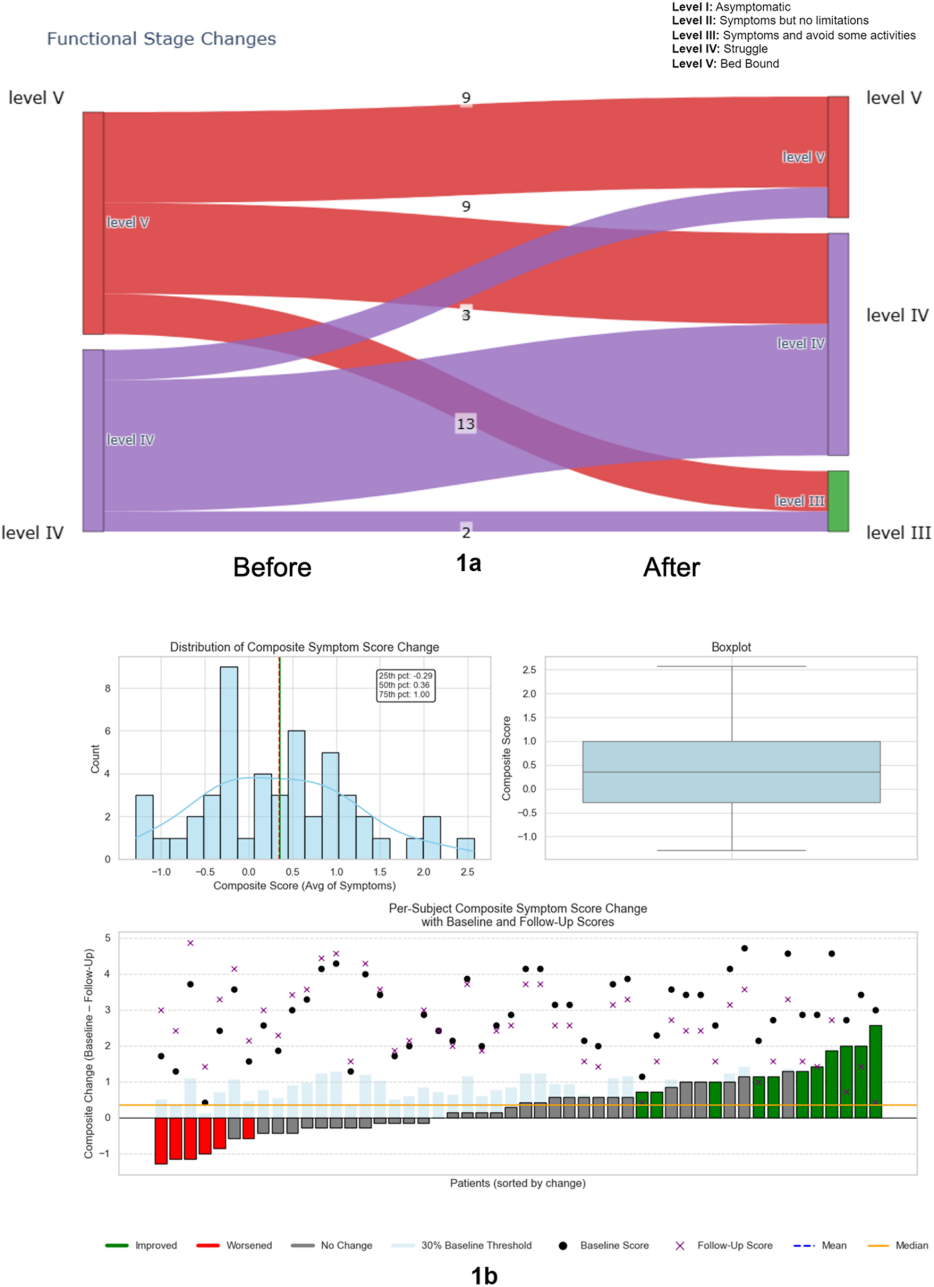
Changes in Functional and Symptom Severity Following Aripiprazole Treatment 1a) Transitions in Functional Status Scale (FSS) before and after Aripiprazole treatment. The Sankey diagram illustrates individual-level changes in functional stage from baseline to last follow-up among 50 patients. Each flow represents the number of patients transitioning between severity levels. 1b) Distribution of composite symptom score changes alongside individual patient trajectories. Among the 50 patients analyzed, 11 (22%) demonstrated a clinically meaningful improvement—defined as a reduction of at least 30% from their baseline composite score—while 6 patients (12%) experienced worsening symptoms beyond the same threshold. The majority (66%) fell within the no-change range. The bar plot highlights this variability, with overlaid markers showing baseline and follow-up scores per subject, and light blue bars indicating each patient’s individualized 30% improvement threshold.

**Table 1.**
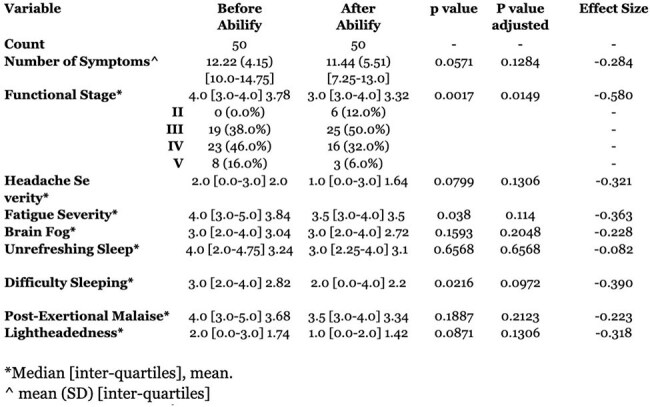
Changes in Symptoms and Function After Aripiprazole Symptoms and Functional Status Scale before and after LDA treatment. p values are adjusted for multiple testing with Benjamini-Hochberg False Discovery Rate.

**Methods:**

A retrospective study using Electronic Health Records (EHR) from 50 adult patients (pts) from the LC clinic with a history of SARS-CoV-2 who received LDA between 5/2021 and 10/2023. LDA was prescribed as a dose-titration (0.1- 2.0 mg daily). We included (i) 29 Sx, (ii) severity of each Sx on the Likert-scale 1 (mild) to 5 (very severe), and (iii) the LC Functional Status (FS) Scale from I (normal) to V (bed-bound). The 7 most common Sx (headache, fatigue, brain fog, unrefreshing sleep, sleep difficulty, post-exertional malaise, and lightheadedness) and a computed composite score (CCS) were assessed pre- and post-treatment using Wilcoxon Signed-Rank Tests. Pts were stratified by Sx duration (< 365 days). Mann-Whitney U, Chi-Square/Fisher’s Exact Tests, and Benjamini-Hochberg correction compared groups. A clinically meaningful improvement was defined as ≥ 30% reduction.

**Results:**

The median age was 45 years, 62% female, and BMI of 28.8 (Table 1). The median duration of Sx was 566.5 days. The FS significantly improved (adjusted p = 0.0149; effect size = –0.580), with reductions in the proportion of pts in severe stages (Figure 1). A reduction in the CCS showed a statistically significant improvement (p < 0.001). Symptom-level analysis revealed moderate improvements in difficulty sleeping (effect size = –0.390), fatigue (–0.363), and headache (–0.321), though none remained significant after multiple testing correction. Eleven pts (22%) exhibited a clinically meaningful ≥ 30% reduction in composite symptom scores. Symptom duration before treatment ( > 365 vs. < 365 days) did not significantly affect treatment response.

**Conclusion:**

LDA was associated with improvements in FS and CCS, and modest improvement in the 7 selected Sx in pts with LC. Treatment with LDA warrants further investigation in a randomized controlled trial as a therapeutic option for LC. Exploring the mechanism of action of Aripiprazole in neuroinflammatory conditions may also provide new insights into the pathogenesis of LC.

**Disclosures:**

Linda Geng, MD PhD, Pfizer: Grant/Research Support

